# Frequency of Antiretroviral Resistance Mutations among Infants Exposed to Single-Dose Nevirapine and Short Course Maternal Antiretroviral Regimens: ACTG A5207

**DOI:** 10.4172/2155-6113.1000371

**Published:** 2014-11-09

**Authors:** Jane Hitti, Elias K Halvas, Lu Zheng, Constantinos G Panousis, Joseph Kabanda, Frank Taulo, Nagalingeswaran Kumarasamy, Jean William Pape, Umesh Lalloo, Heather Sprenger, Karin L Klingman, Ellen S Chan, Deborah McMahon, John W Mellors

**Affiliations:** 1 University of Washington, Seattle, WA, USA; 2 University of Pittsburgh, Pittsburgh, PA, USA; 3 Harvard School of Public Health, Boston, MA, USA; 4 Joint Clinical Res Center, Kampala, Uganda; 5 Queen Elizabeth Ctrl Hospital, Blantyre, Malawi; 6 YRGCARE Medical Centre, Chennai, India; 7 Weill Cornell Medical College, New York, NY and Les Centres GHESKIO, Port-au-Prince, Haiti, Newyork, USA; 8 Durban Adult HIV CRS, Durban, South Africa; 9 Frontier Sci Tech and Research Foundation, Amherst, NY, USA; 10 TRP, DAIDS, NIH, Bethesda, MD, USA

**Keywords:** Infant, HIV-1 Perinatal transmission, Nevirapine, Nucleoside reverse transcriptase, Inhibitors, Antiretroviral resistance, Clinical trials registration: clinicaltrials.gov (NCT00099632)

## Abstract

**Background:**

Intrapartum single-dose nevirapine (sdNVP) reduces HIV-1 perinatal transmission but selects NVP resistance among mothers and infants. We evaluated the frequency of antiretroviral resistance among infants with intrauterine HIV-1 infection exposed to sdNVP and maternal antenatal or breastfeeding antiretroviral therapy.

**Methods:**

This analysis included 429 infants from sub-Saharan Africa, India and Haiti whose 422 mothers received sdNVP plus maternal study treatment. At entry mothers had CD4>250/μL and were ART-naïve except for antenatal ZDV per local standard of care. Maternal study treatment started intrapartum and included ZDV/3TC, TDF/FTC or LPV/r for 7 or 21 days in a randomized factorial design. Infants received sdNVP study treatment and ZDV if local standard of care. Infant HIV RNA or DNA PCR and samples for genotype were obtained at birth and weeks 2, 4 and 12; infants who ever breast-fed were also tested at weeks 16, 24, 48 and 96. Samples from HIV-1-infected infants were tested for drug resistance by population genotype (ViroSeq). NVP or NRTI resistance mutations were assessed using the IAS-USA mutation list.

**Results:**

Perinatal HIV-1 transmission occurred in 17 (4.0%) infants including 12 intrauterine infections. Resistance mutations were detected among 5 (42%) intrauterine-infected infants; of these, 3 had mutations conferring resistance to NVP alone, 1 had resistance to NRTI alone, and 1 had dual-class resistance mutations. Among the 2 infants with NRTI mutations, one (K70R) was likely maternally transmitted and one (K65R) occurred in the context of breastfeeding exposure to maternal antiretroviral therapy.

**Conclusions:**

Infants with intrauterine HIV infection are at risk of acquiring resistance mutations from exposure to maternal antiretroviral medications intrapartum and/or during breastfeeding. New approaches are needed to lower the risk of antiretroviral resistance in these infants.

## Introduction

Although considerable advances have been made in providing potent antiretroviral therapy to all pregnant and breastfeeding women living with HIV, a substantial gap in available antiretroviral coverage still exists. In resource-constrained settings where fully suppressive antiretroviral therapy may not be available for all pregnant women, the World Health Organization currently recommends intrapartum single-dose nevirapine (sdNVP) accompanied by maternal zidovudine and short-course postnatal maternal antiretroviral coverage for women with CD4 cell counts >350 (Option A) [1]. The efficacy of sdNVP depends at least in part on its prolonged half-life which provides detectable NVP concentrations for several weeks after dosing [2]. Prolonged exposure to NVP also selects for NVP resistance mutations in exposed mothers and HIV-1- infected infants [3,4], thus limiting future treatment options for these individuals [5]. Current strategies to prevent the development of new resistance mutations after sdNVP with 7 days of combination antiretroviral therapy still result in emergence of NVP resistance mutations in 12–33% of women [6,7]. In ACTG A5207, the frequency of NVP resistant variants among mothers was reduced to <2% using three different maternal short-course antiretroviral regimens initiated intrapartum and continued for 7–21 days. We now report on the occurrence of HIV-1 MTCT and the frequency of NVP and nucleoside reverse transcriptase inhibitor (NRTI) resistance mutations among infants born to women in ACTG A5207.

## Methods

ACTG A5207 was a phase II, prospective, randomized, open-label study that evaluated the effectiveness of three antiretroviral regimens, initiated intrapartum and continued for 7 or 21 days, to prevent the emergence of new NVP resistance mutations after sdNVP in women. The study design has been previously described in detail [8]. The study population included HIV-1- infected pregnant women and their infants from 8 sites in sub-Saharan Africa, India, and Haiti. The first woman was randomized in January 2007 and the study closed to accrual in January 2010. At entry mothers had CD4 ≥250 cells/uL and were antiretroviral-naïve except for antenatal zidovudine (ZDV) per local standard of care. Prior to onset of labor, women were randomly assigned to receive sdNVP plus either ZDV 300 mg/lamivudine 150 mg (ZDV/3TC) twice daily, tenofovir 300 mg/emtricitabine 200 mg (TDF/FTC) daily, or lopinavir 400 mg/ritonavir 100 mg (LPV/r) twice daily, for either 7 or 21 days, in a factorial design. Randomization was stratified by planned antenatal ZDV. Infants received a single dose of study-supplied NVP within 48 hours of birth. Infants were also allowed to receive ZDV if standard of care, and infants with confirmed HIV infection were allowed to receive other antiretroviral therapy as clinically prescribed. Nevirapine tablets (200 mg) for the mothers and NVP suspension (50 mg/5 mL) for the infants were supplied through the study. Participants were counseled about infant feeding options and those who chose replacement feeding were provided with formula and supplies for water purification for 1 year. The study was approved by sites and their affiliated U.S. partners’ institutional review boards. Written informed consent was obtained from participants.

Infant plasma was obtained for HIV-1 RNA or DNA PCR and stored for future HIV genotyping (if infected) at birth and weeks 2, 4 and 12. Infants with indeterminate HIV-1 status were also tested at weeks 16 and 24. Infants who had ever breast-fed or were HIV-1-infected were also tested at weeks 16, 24, 48 and 96. Infant HIV-1 infection was defined as 2 separate blood samples positive for HIV-1 RNA or DNA PCR. The probable timing of infant HIV-1 infection was characterized as intrauterine if the first positive HIV-1 RNA or DNA occurred within 48 hours of birth. Intrapartum or early breastfeeding was defined by a first positive test after 48 hours and ≤12 weeks of age. Later breastfeeding transmission was defined by a first positive test after 12 weeks of age. The primary HIV-1 infection endpoint was defined as early MTCT, meaning first positive infant HIV-1 RNA or DNA prior to 12 weeks of age.

Plasma HIV-1 DNA and RNA assays for infant HIV diagnosis were performed at ACTG certified laboratories. Plasma samples from HIV-infected infants were tested for antiretroviral drug resistance mutations using the FDA-approved ViroSeq HIV-1 Genotyping System (v.2.0 Celera Diagnostics, Alameda, CA) and performed in batches. Resistance testing was conducted at two ACTG-certified laboratories (Johannesburg, South Africa; Pittsburgh, Pennsylvania USA). Samples that could not be amplified or adequately sequenced using standard ViroSeq primers were tested using Celera’s alternate RTPCR and/or sequencing primers (designated A3, A4, B4, C4, F1, G1, and H1). Resistance testing was not attempted on samples with HIV-1 RNA <400 copies/ml or negative for HIV-1 DNA PCR. All sequencing products were resolved on an ABI 3100 Genetic Analyzer [Applied Biosystems, Foster City, CA] and data was analyzed using ViroSeq v2.7 or Sequencer v4.8 software. NVP or NRTI resistance mutations were defined by the IAS-USA mutation list. All wild type/mutant mixtures were scored as resistant.

Samples positive for the K65R mutation were retested by allele-specific PCR (ASP). The ASP method [7] was modified to include a proofreading polymerase (Phusion, Thermo Scientific) during first round amplification to avoid false positive results arising from errors introduced during PCR amplification [9]. The primers for K65R ASP were, K65wt 5′-CTCCARTATTTGCCATAAAACA-3′, K65R 5′-CTCCARTATTTGCCATAAAACG-3′ and K65REV 5′ TATTCCTAATTGAACYTCCCA-3′. The exact Cochran-Mantel-Haenszel test, Fisher’s exact test and Wilcoxon rank sum test were used to assess statistical significance. All tests were 2-sided at the 5% level of significance with no adjustment for multiple comparisons (SAS v. 9.2, StatXact v. 9).

## Results

**Figure 1:**
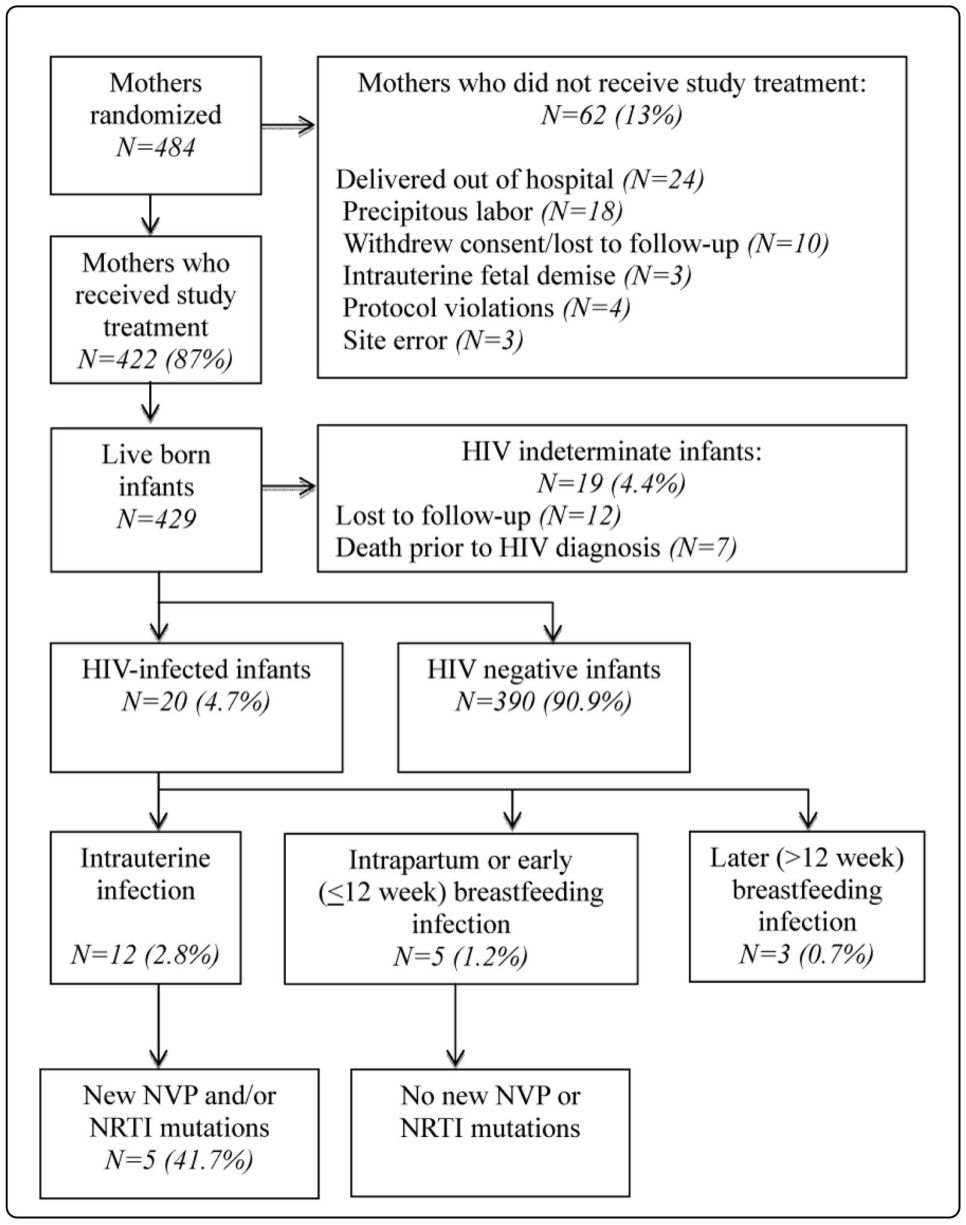
Infant HIV status and detection of NVP or NRTI mutations among study population.

484 women were randomized in ACTG A5207 between January 2007 and October 2009. The current analysis included 429 live infants born to the 422 women who received study treatment. The maternal study population has been previously reported [8]. At study entry, median maternal screening CD4 count was 490 cells/uL and median HIV-1 RNA was 3162 copies/ml. Zidovudine was provided to 63 % of mothers (antenatal) and to 62 % of infants according to local site guidelines. No infants received additional antiretroviral therapy prior to confirmed HIV diagnosis. Median infant birth weight and gestational age at birth were 3000 g and 38 weeks, respectively; 11.9% of infants were low birth weight (<2500 g) and 16.5 % were preterm (<37 weeks). Breastfeeding was reported for 37.5 % of infants (Figure 1).

Figure 1 summarizes infant HIV-1 infection status as well as the detection of antiretroviral resistance mutations. Early MTCT (≤ 12 weeks) occurred in 17 (4.0%) infants and did not appear to be associated with maternal study regimen, treatment duration, antenatal ZDV, birth weight or gestational age. Of these 17 MTCT events, 12 (2.8%) were probable intra-uterine infections and 5 (1.2%) were intrapartum or early breastfeeding infections. Three additional breast-fed infants had HIV-1 infection diagnosed at 15, 36 and 72 weeks of age.

New NVP or NRTI mutations were detected in 5 (42%) of 12 infants with intrauterine HIV-1- infection but were not detected among any of the 5 infants with intrapartum or breastfeeding infection. Among the 12 infants with intrauterine infection, the emergence of resistance mutations did not appear to be associated with maternal entry viral load, screening CD4 count, study regimen, treatment duration, antenatal ZDV; or infant birth weight, gestational age, breastfeeding or ZDV. These new resistance mutations were all detected prior to initiation of other infant antiretroviral therapy beyond sdNVP or ZDV prophylaxis. Four (33%) of 12 intrauterine-infected infants had NVP mutations first detected at weeks 2–4 post-partum. None of their mothers had new NVP mutations detected. Two (17%) of 12 intrauterine-infected infants had NRTI mutations detected at weeks 2–4. Of these, one mother/infant pair was concordant for K70R; the mother received antenatal ZDV and 7 days of LPV/r study treatment, and the infant was not breast fed. The second infant had K65R detected at week 4, after 21 days of maternal study treatment with TDF/FTC; this infant was breast fed. The presence of K65R was confirmed in this infant using allele-specific PCR [7] (Table 1).

Nine (2%) of 429 infants in this cohort died before 12 weeks of age, of whom 2 had intrauterine HIV-1 infection (one with multiple resistance mutations) and 7 had insufficient testing prior to death to determine their HIV-1 status. Early neonatal death was marginally associated with low birth weight (P=.042), and was not associated with maternal study regimen or treatment duration. The predominant causes of early infant death were infections including sepsis (n=3) and gastroenteritis (n=1); two infants died of birth asphyxia and one of respiratory distress syndrome. Overall, the early neonatal mortality was 21 per 1000 live births.

## Discussion

This report underscores concern about the resistance consequences antenatal and/or breastfeeding antiretroviral exposure for HIV-1-infected infants, particularly those who acquire infection prior to birth. In this study, 4 (33%) of 12 intrauterine-infected infants exposed to sdNVP had new NVP resistance mutations detected, consistent with previous studies [3,4]. Intrapartum and maternal postnatal short-course (7–21 days) antiretroviral therapy, such as provided in ACTG A5207, did not protect against the emergence of NVP resistance mutations in intrauterine-infected infants and may have contributed to NRTI resistance in one infant. In total, new NVP and/or NRTI resistance mutations were detected among 5 (42%) of 12 intrauterine infected infants. Such resistance mutations are likely to limit the efficacy of current first-line antiretroviral therapy for these infants.

There may be two mechanisms for the development of new NRTI mutations among intrauterine infected infants in this study. One infant had a new K70R mutation, concordant with a maternal K70R mutation and in the context of antenatal and infant ZDV. These findings suggest that the infant K70R mutation may have been of maternal origin, possibly related to antenatal ZDV exposure. One breast-fed infant had K65R detected at week 4, in the context of maternal treatment with 21 days of TDF/FTC. Presumably, this infant acquired a new K65R mutation after brief intrapartum and/or breastfeeding exposure to maternal study treatment. Tenofovir and emtricitabine have been detected in breast milk in sufficient quantity to allow for infant exposure to these medications [10]. Regardless of the mechanism by which these latter two mutations emerged, their selection may affect the response to antiretroviral therapy.

The main limitation of this study was the small sample size of HIV-1-infected infants for evaluation of resistance mutations. In particular, the number of infants with intrapartum infection (N=5) was too small to draw any conclusions about the emergence of NVP and NRTI resistance mutations in this group. A second important limitation is that the infant plasma volumes were too limited to allow testing for minority variants using more sensitive methods such as allele-specific PCR or single genome sequencing. The detection rate for NVP and NRTI resistance mutations would be expected to be higher with more sensitive methods compared to population genotyping, as reported here.

Lifetime access to potent antiretroviral therapy for all pregnant and breastfeeding women living with HIV (WHO Option B+) is rapidly becoming available in many parts of the developing world. As this access continues to improve, intrauterine HIV transmission will represent an increasing proportion of all pediatric HIV-1-infection; intrauterine infection accounted for 60% of all infant HIV-1 infections in this cohort, including late breastfeeding infection. Thus, the effects of antenatal and breastfeeding antiretroviral exposure on the acquisition of resistance mutations among infants with intrauterine infection should be considered carefully, as should new approaches to preventing resistance. Although the total numbers of HIV-1-infected infants may be expected to decline, the intrauterine infections that occur will undoubtedly be accompanied by new treatment challenges posed by drug resistance.

## Figures and Tables

**Table 1:** New nevirapine and nucleoside reverse transcriptase inhibitor mutations detected among intrauterine-infected infants

Infant	Mutation		Week	Maternal Study Regimen (Duration)	Zidovudine		Breast Fed
	NVP	NRTI			Antenatal	Infant	
1	K103N		2	Lopinavir/ritonavir (7 days)	Yes	Yes	No
	V106A		2				
	Y181C		2,4				
	G190A		2,4				
		K70R*	2				
2	V106A	None	2,4	Lopinavir/ritonavir (21 days)	No	No	Yes
	Y181C		2-97				
3	K103N		4	Lopinavir/ritonavir (21 days)	No	No	Yes
4	V106A	None	4	Lopinavir/ritonavir (21 days)	Yes	Yes	Yes
5	None	K65R		Emtricitabine/tenofovir (21 days)	No	No	Yes

Note: NVP: Nevirapine; NRTI: Nucleoside Reverse Transcriptase Inhibitor;

* Mother concordant for K70R mutation
